# Autohemagglutination Experimentally Induced by the Repeated Withdrawal of Blood

**Published:** 1919

**Authors:** 


***Autohemagglutination Experimentally Induced by the Repeated Withdrawal of Blood.*** By Oswald H. ROBERTSON, M. D., and Peyton ROUS, M. D. *Journal of Experimental Medicine*, May 1, 1918.

The authors have tested the influence of repeated losses of blood on the production of normal isohemagglutinins; this being possibly connected with the remarkable resistance to infection, obvious in some anemic patients.

Experiments were carried out on rabbits; some animals were kept as controls, the remaining ones being bled 10 cc. from the heart every 3 to 6 days, during a period of 2 months.

The results obtained in these experiments were as follows.

No new isoagglutinins developed under the influence of repeated bleedings, nor did normal isoagglutinins already present undergo any changes.

On the other hand, in five out of fourteen bled rabbits, a partial clumping of the red cells occurred in the shed blood. Anemia resulting from malnutrition proved in some cases to have the same effect. This clumping was never detected in the blood of the control rabbits. Only those bearing very poorly the repeated bleedings presented that phenomenon.

The clumps consisted of from 4 to 50 corpuscles massed together. A special agglutinin developing in the serum under the influence of renewed bleedings may be considered as responsible for this clumping. This agglutinin proves to be very active. At 22° C., the serum of bled rabbits was able to bring about clumping in very diluted salt solutions of the animal's washed cells. The serum of normal rabbits tested in the same way never yielded the smallest agglutination.

Changes of temperature do not have the same influence on normal agglutinins and on the agglutinating principle induced by repeated bleedings. The normal agglutinin's power is enhanced when temperature decreases from room temperature to 0° C. The agglutinin's power in bled rabbits, on the contrary, does not seem particularly altered by cooling proceedings. At 0° C. its activity is rather inferior to that of normal agglutinin.

The authors reported that the red cells seemed to lose the tendency to rouleaux formation as the autoagglutinin developed in the serum, thus proving that agglutination and rouleaux formation are not connected, as it was previously believed.

